# Supplementary material for: Holstein-Friesian re-identification using multiple cameras and self-supervision on a working farm
Source: Comput Electron Agric. Author manuscript; Available in PMC 2026 Aug 26. (PMC7619405; doi:10.1016/j.compag.2025.110568)
Supplement: Supplementary file 1 [file EMS217219-supplement-Appendix.pdf]

Appendix

A.1. Dataset metadata

See Figs. A.8 and A.9

A.2. Identifier embedding and metrics visualisation

See Fig. A.10.

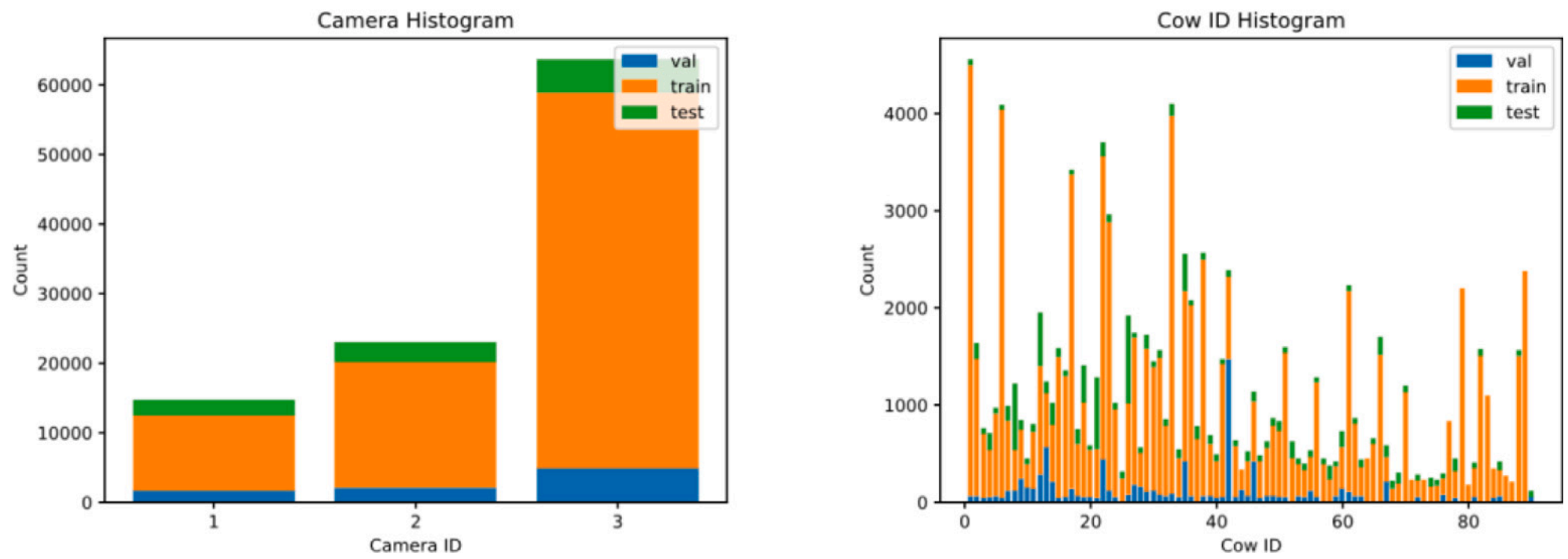

Fig. A.8. Dataset Histogram across Cameras and Individuals. Histogram showing the number of images in our dataset. (Left) Number of Images by camera and (Right) Number of images per ID. Much of the imbalance in numbers is caused by camera 3 which shows a part of the farm where cows are free to stop walking after the milk-race — when this happens, given the fixed sampling rate, many more images are generated.

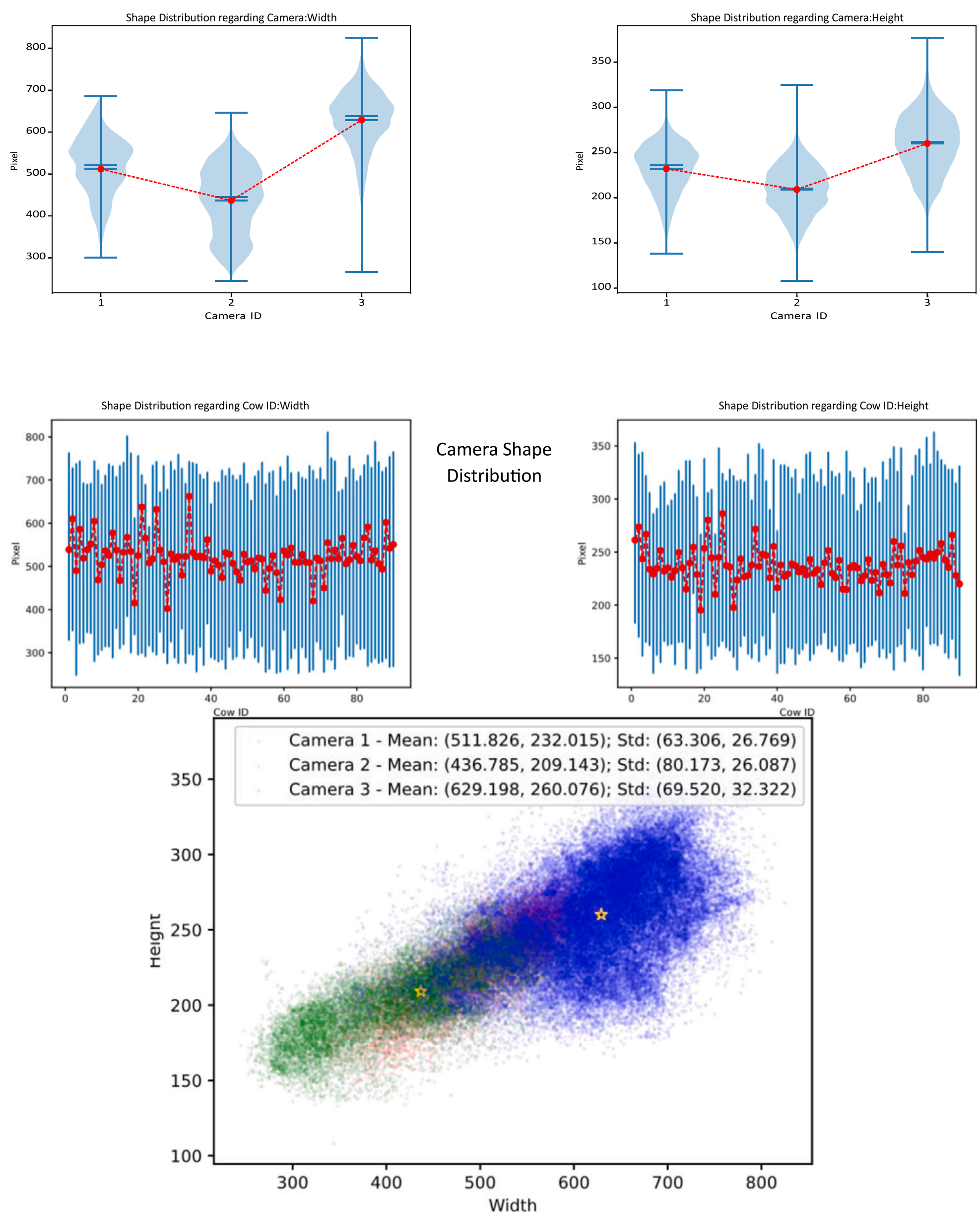

Fig. A.9. Dataset Metadata. Metadata of width and height of cropped cow torsos from our dataset of 90 unique cows. The upper row shows width and height information from different camera views. The middle and lower rows show shape distributions per ID.

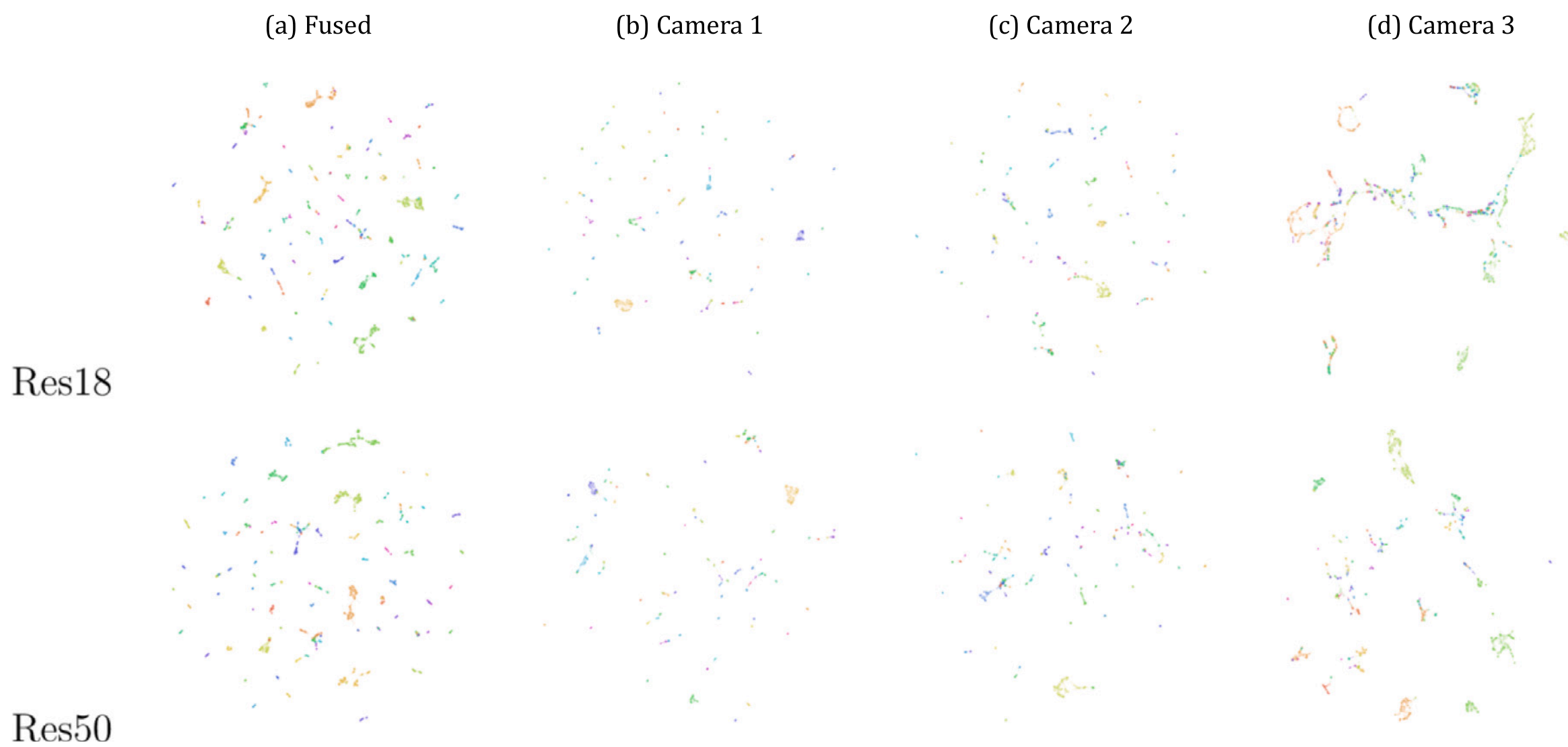

Fig. A.10. Visualisation of Embeddings. Visualisation of embeddings for fused and individual camera perspectives. The false negatives are highlighted whilst dimming the true positives.
